# Plasma Derived Exosomal Biomarkers of Exposure to Ionizing Radiation in Nonhuman Primates

**DOI:** 10.3390/ijms19113427

**Published:** 2018-11-01

**Authors:** Amrita K. Cheema, Charles P. Hinzman, Khyati Y. Mehta, Briana K. Hanlon, Melissa Garcia, Oluseyi O. Fatanmi, Vijay K. Singh

**Affiliations:** 1 Department of Oncology, Lombardi Comprehensive Cancer Center, Georgetown University Medical Center, Washington, DC 20057, USA; akc27@georgetown.edu (A.K.C.); kym8@georgetown.edu (K.Y.M.); 2 Department of Biochemistry, Molecular and Cellular Biology, Georgetown University Medical Center, Washington, DC 20057, USA; cph51@georgetown.edu; 3 Department of Pharmacology and Molecular Therapeutics, F. Edward Hébert School of Medicine, USUHS, Bethesda, MD 20814, USA; brianakh17@gmail.com (B.K.H.); melissa.garcia.ctr@usuhs.edu (M.G.); oluseyi.fatanmi@usuhs.edu (O.O.F.); 4 Armed Forces Radiobiology Research Institute, USUHS, Bethesda, MD 20814, USA

**Keywords:** biomarker, exosomes, gamma-radiation, lipidomes, metabolites, nonhuman primates, plasma

## Abstract

Exposure to ionizing radiation induces a cascade of molecular events that ultimately impact endogenous metabolism. Qualitative and quantitative characterization of metabolomic profiles is a pragmatic approach to studying the risks of radiation exposure since it provides a phenotypic readout. Studies were conducted in irradiated nonhuman primates (NHP) to investigate metabolic changes in plasma and plasma-derived exosomes. Specifically, rhesus macaques (*Macaca mulatta*) were exposed to cobalt-60 gamma-radiation and plasma samples were collected prior to and after exposure to 5.8 Gy or 6.5 Gy radiation. Exosomes were isolated using ultracentrifugation and analyzed by untargeted profiling via ultra-performance liquid chromatography mass spectrometry (UPLC-MS) based metabolomic and lipidomic analyses, with the goal of identifying a molecular signature of irradiation. The enrichment of an exosomal fraction was confirmed using quantitative ELISA. Plasma profiling showed markers of dyslipidemia, inflammation and oxidative stress post-irradiation. Exosomal profiling, on the other hand, enabled detection and identification of low abundance metabolites that comprise exosomal cargo which would otherwise get obscured with plasma profiling. We discovered enrichment of different classes of metabolites including *N*-acyl-amino acids, Fatty Acid ester of Hydroxyl Fatty Acids (FAHFA’s), glycolipids and triglycerides as compared to the plasma metabolome composition with implications in mediation of systemic response to radiation induced stress signaling.

## 1. Introduction

Terrorist attacks with radiological dispersal device (RDD) or improvised nuclear device (IND) weapons are an ever-growing worldwide concern in government and public sectors as they become more violent and more sensational. Their goals are to maximize psychological and economical damage and mortality to the exposed victims [1]. In addition, there is always possibility of radiological or nuclear accidents. In the event of an RDD or IND, a deliberate attack by terrorists, or a nuclear power plant accident, a large number of people may be exposed to high doses of ionizing radiation. Some victims may be asymptomatic while others may exhibit mild to severe symptoms, resulting in death in some cases. 

It is a challenging task for medical responders to triage ionizing radiation-exposed victims into definable, treatment-susceptible groups in a mass casualty scenario. Minimally-exposed (<2 Gy) individuals may not require immediate care, while victims with exposure to moderate (2–6 Gy) or high (6–10 Gy) doses of radiation can most likely benefit from timely treatment. However, those who have received supralethal doses (>10 Gy) of radiation need only palliative care as they cannot recover from these high doses. Identifying these subcategories is essential to conserve the scarce resources during any mass casualty scenario. The currently available strategies to assess the status of individuals exposed to various doses of radiation are usually based on the exposed victim’s signs and symptoms developing over time, or biological dosimetry such as dicentric assay and lymphocyte depletion kinetics. However, dicentrics and lymphocyte kinetic assays are time-consuming, labor-intensive, require well trained staff and are difficult to execute in a mass casualty scenario. Therefore, it is important to develop appropriate biomarkers for radiation exposure based on molecular changes such as proteins and metabolites [2,3].

According to the United States Food and Drug Administration (US FDA), a biomarker can be measured and can indicate a specific biological, pathological, or therapeutic process [4]. The biomarker may reflect biological processes closely related to the mechanism of disease, or a downstream step of the initial indication. These biomarkers can be used to assess different types of biological characteristics. A composite biomarker panel consists of several individual molecules or cellular changes that are combined in a specified algorithm to reach a single output. Robust and compelling scientific evidence is needed to validate a biomarker panel that can be used to stratify individuals at risk of exposure to ionizing radiation. The FDA has further defined its qualifications to validate a biomarker by the ability to reliably and reproducibly measure the biomolecules of interest [5]. Biomarkers are used in the diagnostic, prognostic, predictive and pharmacodynamic processes of drug development. A single biomarker may play a role in more than one step of the drug developmental or injury assessment. Biomarkers are an important aspect of radiation countermeasure development and used as a trigger for intervention, in selecting a drug dose and treatment schedule. Biomarkers may correlate with the mechanism by which the agent reduces the injury inflicted or used to correlate the desired clinical outcome. The nonhuman primate (NHP) model most closely reproduces the histopathological, clinical and pathophysiological attributes of radiation injury in humans [6]. In addition, because of their longer life span and similar supportive care requirements for acute radiation syndrome, it is possible to link the dose effect relationships between NHP models and humans following the known medical management and treatment. Several promising pharmaceutical agents are under advanced development as radiation countermeasures following the FDA Animal Rule. Efficacy studies for these drugs are currently under investigation in our laboratory.

Exosomes are formed from intraluminal vesicles and are delivered from multi-vesicular bodies to the outside of the cell by fusion with the extracellular membrane [7,8]. Exosomal cargo contains proteins, nucleic acids, lipids, miRNAs and metabolites deemed to facilitate cell-to-cell communication under normal and diseased conditions [9,10]. Exosomes are a rich untapped resource for discovering novel biomarkers that can be leveraged as biomarkers of radiation exposure and offer novel insights into possible mechanisms of radiation injury. Not surprisingly, exosomal research has been increasingly gaining credence as exosomes have been proven to be carriers of important cellular information which can be used as a readout of response to genetic or environmental perturbations [7]. We have optimized an experimental pipeline able to be broadly applied for the identification of low abundance biomarkers from plasma samples [11,12]. We have also shown that several biomolecules identified via plasma exosomal profiling are below limits of detection when profiling intact plasma; thus, justifying the need to enrich this fraction for MS analyses.

In this study, we present the metabolic profile in plasma and plasma-derived exosomes of NHPs exposed to 5.8 (LD_20−40/60_) or 6.5 Gy (LD_30−50/60_) total-body ^60^Co γ-radiation. ANOVA comparison within each radiation dose group was performed to identify features that were dysregulated over time. Binary Student’s *t*-tests were also performed between controls and irradiated NHPs, for each of the time points in each radiation group. Significantly dysregulated features were annotated using MS/MS and SIMLIPID software, via fragmentation pattern matching. Our results show that enrichment of the exosomal fraction of plasma adds value to metabolomics-based biomarker discovery since it enables detection of low abundance metabolites. To our knowledge, this is the first report on radiation metabolomics in exosomes using NHPs as a model system.

## 2. Results

### 2.1. Exposure to Ionizing Radiation Enhances Exosomal Shedding in Circulation

**Figure 1:**
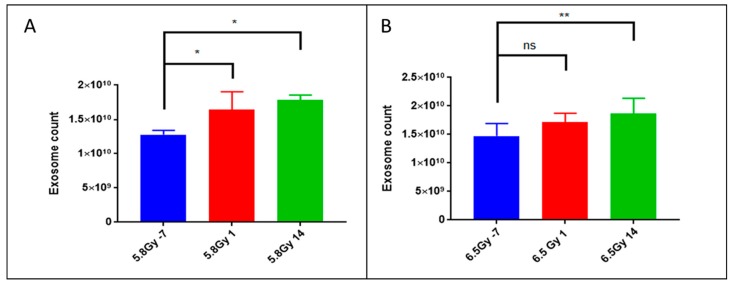
5.8 Gy and 6.5 Gy irradiation exposure results in significantly increased exosomal shedding. Using quantitative ELISA targeting CD63, a well-known marker for exosomes, plasma-derived exosome samples were quantified. After exposure to ^60^Co γ-radiation, an increase in the number of isolated exosomes was noted. Exosome counts at 7-d prior, 1 d post- and 14 d post-irradiation with (**A**) 5.8 Gy or (**B**) 6.5 Gy ^60^Co γ-radiation. *p*-values: ns = not significant, * = <0.05, ** = <0.01.

Exosomes are membrane enclosed vesicles ranging from 30 to 100 nm in size, derived from endocytic multivesicular bodies and secreted by most tissue and organ types in biofluids and into circulation [13,14]. We previously published one of the first reports on optimization of exosome isolation from biofluids as well as from cell culture supernatant for metabolomics and lipidomics characterization [11]. Recently, several groups have shown the importance of characterizing exosomes for gaining novel insights into the onset and progression of different pathologies [15,16,17]; however, the utility of this approach is yet to be explored in radiation research. Herein, we enriched the exosomal fraction of plasma samples obtained from NHPs before (−7 d) and after (d 1 and d 14) exposure to 5.8 or 6.5 Gy γ-radiation. To validate successful enrichment of the plasma exosomal fraction, we quantified the total number of exosomes in each sample using an ELISA specially designed to target CD63 [18]. Interestingly, we found that the number of exosomes per µL of plasma increases significantly by d 1 post-irradiation with 5.8 Gy (Figure 1A) and d 14 post-irradiation with both doses (Figure 1A,B). This is a striking and novel finding that radiation exposure increases secretion of exosomes in the circulation and this corroborates with other studies [19,20]. 

### 2.2. Metabolomic and Lipidomic Profiles of Plasma and Plasma-Derived Exosomes

**Figure 2:**
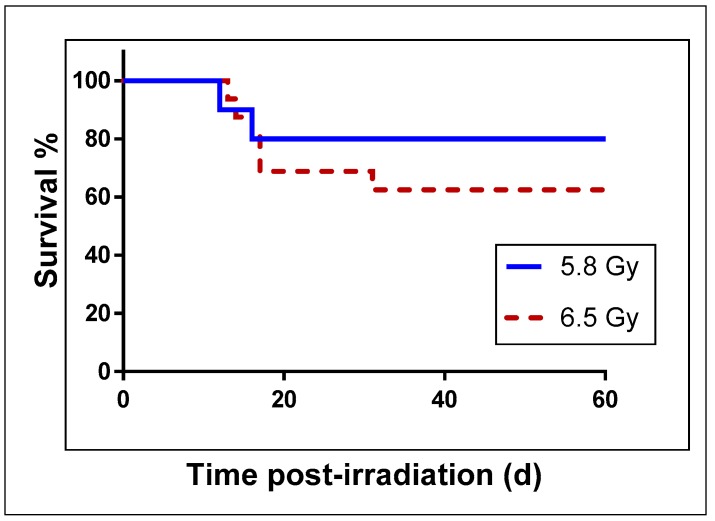
Survival curve for nonhuman primates (NHPs) exposed to 5.8 Gy (LD_20−40/60_**)** or 6.5 Gy (LD_30−50/60_) total-body ^60^Co γ-radiation. NHPs were exposed to a specific radiation dose at a dose rate of 0.6 Gy/min (bilateral, simultaneous) and observed for 60 d for survival.

NHPs were exposed to either 5.8 Gy (LD_20−40/60_, *n* = 10) or 6.5 Gy (LD_30−50/60_, *n* = 16) total-body ^60^Co γ-radiation. The two groups showed a difference in survival. Kaplan Meier survival curves presented in Figure 2 demonstrate that the group receiving 5.8 Gy had 80% survivors while the group exposed to 6.5 Gy had 62% survivors. Since we usually use these two radiation doses for investigating efficacy of radiation countermeasures for hematopoietic acute radiation syndrome (H-ARS) in NHPs, we were interested to study the metabolomics profiles in such animals and compare pre- versus post-irradiation samples. 

**Figure 3:**
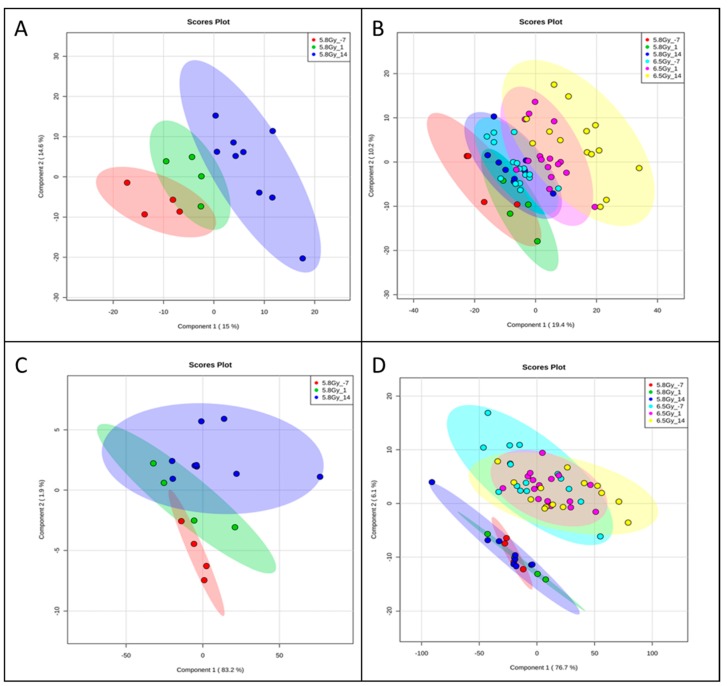
Group separation by total features in (**A**) plasma and (**B**) plasma-derived exosomes. Partial least squares discriminant analysis (PLS-DA) plots demonstrating separation by time and dose in plasma and plasma-derived exosome samples. All identified features were used. Though both plasma and exosomes were able to reasonably separate between pre- and post-irradiation, exosome feature profiles were superior at separating between doses. (**A**) 5.8 Gy plasma, (**B**) 5.8 Gy and 6.5 Gy plasma, (**C**) 5.8 Gy exosomes, (**D**) 5.8 Gy and 6.5 Gy exosomes. Representative data from electrospray positive (ESI+) ionization mode.

In order to understand the potential use of exosomes in investigating biomarkers of radiation damage, plasma was collected from these NHPs. After isolating exosomes from plasma, we performed UPLC/quadrupole time-of-flight (QTOF)-MS to characterize exosome and plasma metabolomic profiles. Pre-processing the raw LC-MS data through XCMS identified a total of 2689 features in electrospray ionization (ESI) positive mode and 3658 in ESI negative mode, in plasma. In the plasma-derived exosomes, we identified a total of 2302 features in ESI positive mode and 2470 features in ESI negative mode. All features were subjected to multivariate analyses including partial least squares discriminant analysis (PLS-DA) to ascertain separation between groups. Examination of the PLS-DA plots for a time dependent comparison for a given dose (5.8 or 6.5 Gy) yielded good separation for exosomes as well as plasma (Figure 3A,C) (R^2^ = 0.89, Q^2^ = 0.49 across 2 components for plasma and R^2^ = 0.78, Q^2^ = −0.35 across 2 components for exosomes). Interestingly, the feature composition of the exosomal fraction was able to discriminate the samples based on the radiation doses with much higher clarity as compared to plasma from the same animals (Figure 3B,D, R^2^= 0.60, Q^2^ = 0.52 across 2 components for exosomes and R^2^ = 0.74, Q^2^ = 0.56 across 2 components for plasma). 

**Figure 4:**
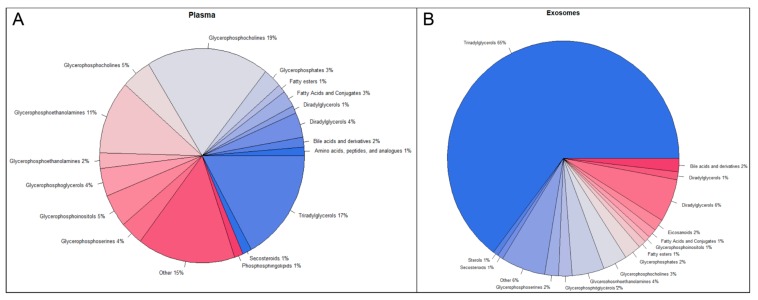
Compound profiles of exosomes and plasma based off accurate mass database search. All significant *m*/*z*’s for both plasma and plasma-derived exosomes were subjected to accurate mass database search through CEU-MassMediator, the Human Metabolome Database (HMDB) and Metlin. Potential identities for each *m*/*z* were screened for their biological relevance. Class for each potential identification was then pulled from the respective database and graphed as a pie chart to help visualize potential metabolite and lipid profile differences between (**A**) plasma and (**B**) exosomes.

Statistical analyses identified 942 *m*/*z*’s which were statistically significantly dysregulated at 5.8 Gy and 1544 at 6.5 Gy. For the exosomal fraction, we found a total of 3505 statistically significantly dysregulated *m*/*z*’s at 5.8 Gy and 3915 *m*/*z*’s at 6.5 Gy. To better understand the differences between plasma and plasma-derived exosomes, we subjected the significantly dysregulated *m*/*z*’s for each matrix to accurate mass-based database search using CEU-MassMediator, the Human Metabolome Database (HMDB) and Metlin. We classified each *m*/*z* as biologically relevant, or not and obtained the formal class information for potential identification of each *m*/*z*, to determine the small molecule profile for whole plasma (Figure 4A) as compared to the enriched exosomal fraction (Figure 4B). Not surprisingly, we found that most of the *m*/*z*’s in both the exosomes and in plasma were likely lipids (primarily triacylglycerols and glycerophospholipids). Interestingly, however, the exosomes seemed to be enriched significantly for glycerolipids (16% as compared to 8% in plasma), fatty acids (8% compared to 4% in plasma) and triglycerides (8% compared to 2% in plasma) (Figure 4B), a phenomenon we have observed in separate, unrelated studies (data not shown). In addition, the exosomal fraction was significantly enriched in bile acids and derivatives, eicosanoids, diradylglycerols, and fatty acid conjugates as compared to plasma. 

### 2.3. Exosomes Yield Metabolic Information that May Be Missed from Plasma Profiling 

**Figure 5:**
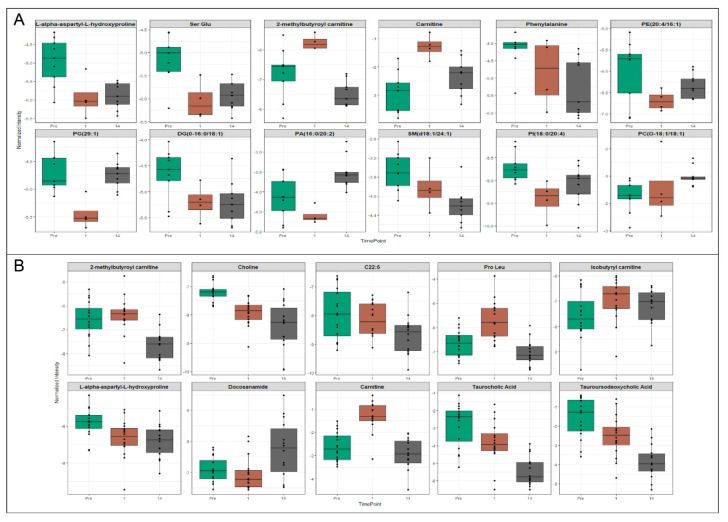
Significantly dysregulated biomarkers in plasma of NHPs exposed to ^60^Co γ-radiation. Select biomarkers that were putatively annotated in plasma samples from NHPs exposed to either 5.8 Gy (**A**) or 6.5 Gy (**B**) ^60^Co γ-radiation. Potential biomarkers for radiation damage were significantly changed and unique to plasma profiling including Carnitine, Ser-Glu, Choline, isobutyryl carnitine and L-alpha-aspartyl-L-hydroxyproline.

**Figure 6:**
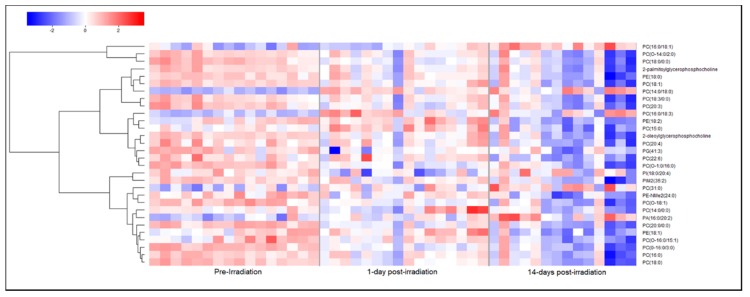
NHPs exposed to 6.5 Gy ^60^Co γ-radiation experience significant dyslipidemia in plasma. Heat map visualizing an initial up-regulation of a significant number of lipid classes 1-d post-irradiation in NHP plasma samples. Nearly all lipids that were significantly changed were down-regulated after 14-d post-irradiation when compared to pre-irradiation levels, suggesting an initial increase in lipid synthesis followed by a delayed systemic injury response.

Based on the differences in metabolite composition between plasma and exosomes, it appears that exosomal profiling would augment the identification of low abundance biomarkers which would otherwise be obscured in plasma or would require large amounts of plasma to be processed for LC-MS based profiling. Therefore, we compared potential biomarkers that were putatively annotated in plasma to those that were putatively annotated in exosomes. Of the total significantly dysregulated *m*/*z*’s, we validated 52 metabolites in plasma at radiation doses of either 5.8 Gy (12) or 6.5 Gy (40) and 18 metabolites in exosomes at either 5.8 Gy (9) or 6.5 Gy (9), using tandem MS. At a dose of 5.8 (Figure 5A) and 6.5 Gy (Figure 5B), the annotated biomarkers in plasma included carnitines, lipids including phosphatidic acid, sphingomyelins, phosphatidylinositol, phosphatidyl ethanolamine and glycerophosphocholines and the amino acid phenylalanine. Most of these metabolites showed a decline in plasma levels post-irradiation (d 1 and d 14) although some metabolites reverted to near normal levels on d 14 post-irradiation. Additionally, we found significant lipid dysregulation in the 6.5 Gy cohort plasma samples; interestingly, most lipid classes were found to be moderately up-regulated on d 1 post-irradiation but by d 14 expression levels significantly dropped off compared to pre-irradiation levels (Figure 6). 

**Figure 7:**
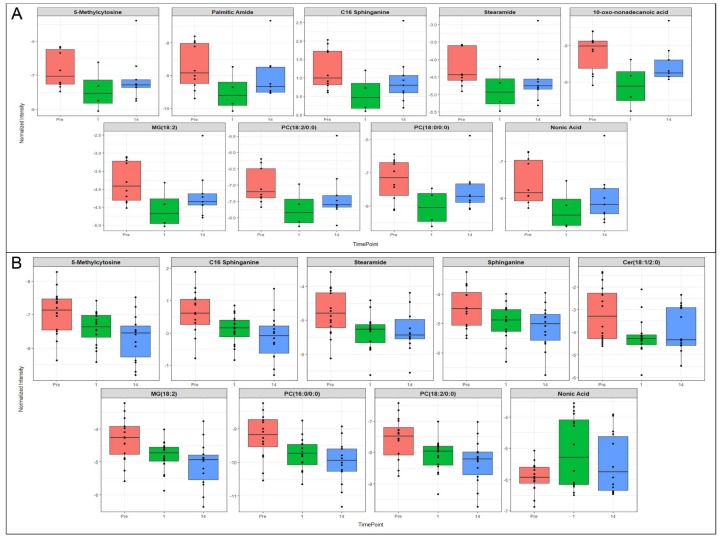
Biomarkers of interest significantly dysregulated in plasma-derived exosomes from NHPs exposed to ^60^Co γ-radiation. Select metabolites putatively annotated only in exosome samples from NHPs exposed to either 5.8 Gy (**A**) or 6.5 Gy (**B**) ^60^Co γ-radiation.

A similar investigation of exosomal profiles at 5.8 Gy (Figure 7A) and 6.5 Gy (Figure 7B) showed down-regulation of glycerophospholipids (similar to the trend seen in plasma). However, we validated several metabolites specific to exosomes including 5-methycytosine, palmitic acid, C16 sphinganine and Nonic acid. Nonic acid is an anionic form of succinate and serves as a dicarboxylic group that can be transported through the mitochondrial matrix and hence dysregulation of this metabolite is suggestive of mitochondrial dysfunction, while changes in 5-methycytosine levels are suggestive of epigenetic changes in response to radiation exposure that are likely to impact downstream gene expression [21].

## 3. Discussion

In a scenario where people do not show up to the hospital within 24 h of a radiation fall out event, it is imperative that the predictive markers be stable over time, thus enabling radiation exposure assessment. Additionally, a systematic undertaking aimed at characterizing systemic changes in response to specific exposures would facilitate better clinical management of “at-risk” individuals. Despite several studies, a specific biomarker panel for stratifying individuals at risk of exposure to radiation has not been well delineated [2,3,22,23,24]. Most biodosimetry studies have used qualitative approaches and need to be validated and verified in higher-order model systems. We and several others have published extensively on radiation biomarkers using metabolomics and lipidomic approaches with different doses of radiation and measurements at various time points post-exposure [23,24,25,26,27]. Identification of sensitive and specific biomarkers with clinical and translational utility, will require smart experimental strategies that would augment expanding the breadth and depth of molecular measurements within the constraints of currently available technologies. In this study, we used two different doses of ^60^Co total-body γ-radiation where animals received bilateral simultaneous exposure with 5.8 Gy (LD_20−40/60_) or 6.5 Gy (LD_30−50/60_). Any dose of total-body irradiation above 5.0 Gy is lethal in NHPs without supportive care (blood product transfusion and use of antibiotics based on culture sensitivity). Both of the doses used in this study induce H-ARS in NHPs with significant cytopenia, neutropenia and thrombocytopenia within two weeks of radiation exposure which makes these doses an optimal choice for investigating efficacy of promising radiation countermeasures [28]. These doses of total-body γ-irradiation in NHPs are comparable to 3.5–4.5 Gy exposure dose for humans (approximate LD_50_ value). 

Radiation metabolomics has not only contributed to the development of biomarkers in minimally invasive matrices including plasma and urine but also paved novel insights into systemic dissemination of radiation response [23]. More recently, the concept of exosomes as systemic mediators of stress response has gained increasing credence in the context of radiotherapy [29,30]. However, exosomal metabolomics is yet to be explored for delineating novel biomarkers that would otherwise be obscured with plasma profiling. Kulkarni et al. have recently reported on exosome associated proteomic biomarkers in mice exposed to sub-lethal doses of whole-body radiation in urine and plasma [31]. They found a time dependent proteomic signature suggestive of inflammation and vascular injuries. Herein, we have used high resolution MS in conjunction with UPLC to characterize changes in small molecule abundance in NHPs before (−7 d) and after exposure (d 1 and d 14) to two different doses of ionizing radiation (5.8 Gy and 6.5 Gy) which induces H-ARS in NHPs. 

We observed that radiation exposure increased secretion of exosomes in the circulation which corroborates with other studies demonstrating increased exosome production and/or excretion in other pathophysiological conditions [19,20]. Moreover, since exosomes are known to have cell to cell communicative properties, this could suggest mediation of radiation stress signaling via exosomal secretion. As expected, radiation exposure-induced a significant systemic response at both doses. The feature composition of the exosomal fraction was able to discriminate samples based on the radiation doses with much greater clarity as compared to plasma from the same animals (Figure 3B,D). We found an increase in carnitines at 24 h with plasma profiling, a finding that we have also reported with serum metabolomics profiling in NHPs [32]. However, the levels stabilize by d 14 post-irradiation. We found a decrease in monoacylglycerol (MG 18:2) on d 1 and d 14 after irradiation. Although some studies have reported an increase at 24 h post-irradiation that is suggestive of acute effect of irradiation [32].

On the other hand, enrichment of the exosomal fraction led to complimentary coverage of the metabolome with significant changes in several metabolites including 5-methyl cytosine, nonic acid, ceramides and sphinganines as compared to pre-exposure levels. These metabolites have important roles in cell-cell signaling, communication and regulation of apoptosis and could potentially be mediators of radiation response. We found that the metabolites which are unique to exosomes are not significantly changed and/or detectable in plasma. While this could be due to enhanced stability perpetuated by exosomes, it is likely that the detection of these metabolites in exosomes is simply due to enrichment, whereas they are masked in total plasma profiling or below the limits of detection. On the other hand, for overlapping metabolites in the two matrices, we did not find enhanced stability in the exosomal fraction as compared to plasma (data not shown). The enrichment of triglycerides in the exosomal fraction is an interesting finding, given the role of this class of metabolites in perpetuating oxidative stress. Further, the exosomal fraction was significantly enriched in bile acids and derivatives, eicosanoids, diradylglycerols, and fatty acid conjugates as compared to plasma. These classes of metabolites including bioactive lipids have been shown to be associated with radiation induced stress signaling [33,34]. Based on the differences in metabolite composition between plasma and exosomes, we suggest that exosomal profiling would augment the identification of low abundance biomarkers that would otherwise be obscured in plasma or would require large amounts of plasma to be processed for LC-MS based profiling. These results warrant further investigations into possible functional and signaling roles that exosomes may have in mediating systemic effects of radiation exposure. The findings reported here also reveal a new avenue for research aimed at understanding the molecular basis of radiation response mediated by circulating exosomes.

## 4. Materials and Methods

### 4.1. Animals and Animal Care

Twenty-six naïve rhesus macaques (*Macaca mulatta*, Chinese sub-strain, 11 males and 15 females) 3–6 years of age, weighing 4 to 8 kg, were obtained from the National Institutes of Health Animal Center (NIHAC, Poolesville, MD, USA) and maintained in a facility accredited by the Association for Assessment and Accreditation of Laboratory Animal Care (AAALAC)-International. Animals were quarantined for six weeks prior to initiation of the experiment. Animal housing, health monitoring, care and enrichment during the experimental period have been described earlier [35]. All procedures involving animals were approved by the Armed Forces Radiobiology Research Institute Institutional Animal Care and Use Committee (IACUC) and Department of Defense Animal Care and Use Review Office (ACURO) (AFRRI IACUC protocol # P2015-01-001, approval date 30 March 2016). This study was carried out in strict accordance with the recommendations in the *Guide for the Care and Use of Laboratory Animals of the National Institutes of Health* [36].

### 4.2. Radiation Exposure

NHPs were exposed to either 5.8 or 6.5 Gy total-body ^60^Co γ-radiation (dose rate 0.6 Gy/min, bilateral simultaneous exposure) as described earlier [28]. Dose rate measurements were based primarily on the alanine/EPR (electron paramagnetic resonance) system as described earlier [28].

### 4.3. Plasma Sample Collection

Blood was collected by venipuncture from the saphenous vein of the lower leg and plasma was separated as previously described [33]. 

### 4.4. Exosome Isolation and Characterization

Exosomes were isolated by adapting our previously described methodology [11]. Briefly, 100 µL of NHP plasma was diluted in 30 mL of 1X PBS in a 50 mL conical tube. Samples were centrifuged at 1600× *g* for 20 min to separate cell debris and other macromolecules. Supernatant was transferred to ultracentrifuge tubes, balanced with 1X PBS and centrifuged using an SW-28 swing bucket rotor (Beckman Coulter, Indianapolis, IN, USA) in an Optima XE Ultracentrifuge (Beckman) at 10,000× *g* for 20 min at 4 °C. Supernatant was then filtered, using a syringe, through a 0.2 µm filter into new ultracentrifuge tubes. Samples were then re-balanced with 1X PBS and ultracentrifuged at 120,000× *g* for 60 min at 4 °C. Supernatant was carefully aspirated and exosomal pellets were re-suspended in 50 µL of 1X PBS and stored at −80 °C for further analysis. Quantification of exosomes was performed using the ExoELISA-ULTRA Complete Kit with CD63 detection (System Biosciences, Palo Alto, CA, USA), according to the manufacturers’ protocol. Exosome counts were used to normalize MS intensities for each detected *m*/*z*. 

### 4.5. Sample Preparation for Mass Spectrometry (MS)

Plasma samples were prepared as described previously [37]. Briefly, 25 µL of NHP plasma was mixed with 75 µL of 40% isopropanol +25% methanol +35% water containing internal standards (4-nitrobenzoic acid and debrisoquine). Samples were vortexed, incubated on ice for 20 min and combined with 100 µL of ice-cold acetonitrile. Samples were vortexed again, incubated at −20 °C for 15 min, centrifuged at 13,000 rpm for 20 min at 4 °C and supernatant was transferred to MS vials for analysis.

Exosome samples were similarly prepared for MS analysis as previously published [11], with slight modifications. Exosomes re-suspended in 50 µL 1X PBS were placed on dry-ice for 30 s and heat shocked in a 37 °C water bath for 90 s. This was repeated three times. Samples were then sonicated for 30 s, vortexed and incubated on ice for 20 min and mixed with 150 µL of ice-cold internal standard solution (as described above) and 150 µL of ice-cold acetonitrile was added to each sample. Samples were again vortexed and incubated at −20 °C for 25 min. After incubation, samples were centrifuged at 13,000 rpm for 20 min at 4 °C and supernatant was transferred to mass spectrometry vials for data acquisition.

### 4.6. UPLC/Q-TOF-MS Data Acquisition and Statistical Analysis

Untargeted metabolomics and lipidomics data was acquired as previously described for plasma [37] and exosomes [11]. Raw MS data were pre-processed using XCMS (a web-based platform to process untargeted metabolomic data, Scripps Research Institute, La Jolla, CA, USA) in “R.” Intensities were normalized to internal standards and for exosome samples, exosome concentration. Multivariate statistical analyses were performed using Metaboanalyst V4.0 [38] with log transformation and Pareto scaling. Statistically significant *m*/*z*’s, determined by Student’s two-tailed *t*-test or ANOVA (FDR adjusted *p*-values < 0.05), were subjected to database search for identification and biological relevance using Metlin [39], CEU Mass Mediator [40] and HMDB [41]. Metabolites and lipids were subsequently putatively annotated via Tandem MS and SimLipid V6.0 (Premier Biosoft, Palo Alta, CA, USA), respectively. Figures were generated using Metaboanalyst, GraphPad Prism 7 (GraphPad Software, La Jolla, CA, USA) and custom R scripts.
